# Robust water desalination membranes against degradation using high loads of carbon nanotubes

**DOI:** 10.1038/s41598-018-21192-5

**Published:** 2018-02-09

**Authors:** J. Ortiz-Medina, S. Inukai, T. Araki, A. Morelos-Gomez, R. Cruz-Silva, K. Takeuchi, T. Noguchi, T. Kawaguchi, M. Terrones, M. Endo

**Affiliations:** 1 Global Aqua Innovation Center, Shinshu University, Nagano, 380-8553 Japan; 2 Division of Computational Science and Technology, Research Organization for Information Science and Technology, Tokyo, 140-0001 Japan; 3 Institute of Carbon Science and Technology, Faculty of Engineering, Shinshu University, Nagano, 380-8553 Japan; 4 Department of Physics, Department of Chemistry, Department of Materials Science and Engineering & Center for 2-Dimensional and Layered Materials, The Pennsylvania State University, University Park, PA 16802 U.S.A.

## Abstract

Chlorine resistant reverse osmosis (RO) membranes were fabricated using a multi-walled carbon nanotube-polyamide (MWCNT-PA) nanocomposite. The separation performance of these membranes after chlorine exposure (4800 ppm·h) remained unchanged (99.9%) but was drastically reduced to 82% in the absence of MWCNT. It was observed that the surface roughness of the membranes changed significantly by adding MWCNT. Moreover, membranes containing MWCNT fractions above 12.5 wt.% clearly improved degradation resistance against chlorine exposure, with an increase in water flux while maintaining salt rejection performance. Molecular dynamics and quantum chemical calculations were performed in order to understand the high chemical stability of the MWCNT-PA nanocomposite membranes, and revealed that high activation energies are required for the chlorination of PA. The results presented here confirm the unique potential of carbon nanomaterials embedded in polymeric composite membranes for efficient RO water desalination technologies.

## Introduction

Current technologies for large-scale water desalination from seawater or brackish water are mainly based on reverse osmosis (RO) membranes, which have been improving since the 1960s^1^. The construction of RO membranes relies mostly on thin film composite (TFC) systems, in which the active layer is a polymer film deposited on top of a highly permeable porous substrate. TFC membrane’s active layers are responsible for solvated ions rejection, and nowadays they are usually made of aromatic polyamides (PA). Given the importance of RO-based purification systems, a considerable effort has been focused on reducing operational costs and energy consumption in water desalination plants. Therefore, it is very important to develop novel RO membranes with improved water permeability and salt rejection within the active layer, being robust against chemical and biological degradation processes. In this context, a viable alternative is the incorporation of carbon nanotubes (CNT) into the polymer matrix to form polymer nanocomposite films^2–4^ or even using carbon nanotube-based membranes^5,6^.

Nowadays, chlorine degradation is one of the major drawbacks that reduces the operation lifetime of PA-based RO membranes, since chlorine compounds are usually added to the feed water for preventing biofouling in water desalination processes, or for disinfection in food separation systems. PA deterioration by chlorine results in the passage of salt and water, thus reducing the membrane performance lifetime; the underlying mechanism for the chlorine induced degradation has been studied for several years^7–9^. Most of the proposed pathways involve several chemical steps and structural rearrangements that include a reversible and irreversible chlorination^10^, with few factors that are found to be critical for PA membrane degradation, such as free chlorine concentration, pH and exposure time. The latest research indicates that the degradation process is mainly due to the amide bond scission, produced after the chlorination of the nitrogen at the amide link^9,11^. In fact, the complete process is divided into two phases^12^: chlorine uptake, strongly depending on the free chlorine concentration, and a subsequent amide bond scission facilitated by the presence of hydroxyl ions (OH^−^). The incorporation of low amounts of carbon nanomaterials such as CNT or graphene to the composite membranes has been already proposed as an alternative for increasing their resistance to chlorine degradation^2,3,13^. However, the mechanisms for a decreased PA degradation in the presence of different amounts of carbon nanomaterials loads still need to be understood in detail. We recently reported a TFC RO membrane with an active layer made of a MWCNT-PA nanocomposite film^14^. The active layer contained *ca*. 15 wt.% of nanotubes with diameters ranging between 12 and 15 nm, and we found that MWCNT not only improved the membrane performance in terms of water flow, salt rejection and biofouling resistance compared with other composite RO membranes^2,3^, but also increased its tolerance to chlorine exposure. We proposed that the PA matrix adopted a distinctive structure, in which the first two layers of aromatic moieties were preferentially oriented around the nanotubes^15^. Such polymer nanostructures combined with rigid CNT result in a more robust membrane when compared to plain PA. Upon hydration, the PA matrix of these nanocomposite membranes is mechanically constrained, thus a low fraction of large voids that can accommodate water pockets, are formed. Consequently, CNT reinforced PA membrane shows lower absorption of chlorine when compared to plain PA as shown by XPS analysis^16^.

In the present paper, we study the chlorine resistance performance of MWCNT-PA RO composite membranes, by evaluating for the first time their water flux and salt rejection properties before and after exposure to aqueous sodium hypochlorite (NaClO) solutions as a function of MWCNT load (from plain PA to 20 wt.% MWCNT load), using harsh operation conditions comparable to industrial processes (i.e. high salinity water separation tests under high pressure cross-flow conditions). Furthermore, computer simulations of MWCNT-PA were used to elucidate the resistance mechanism of chlorine within these composite membranes. The obtained results demonstrate a feasible protective mechanism attributed to the presence of an increased amount of MWCNT within the PA matrix, and to the stabilizing effect of large diameter MWCNT which hinder the chlorination of the PA structure. Thus, MWCNT-PA composite membranes offer an alternative route for improving the performance of current RO technologies, especially in applications where membrane robustness against chlorine degradation is critical.

## Results and Discussion

### Structural characterization

**Figure 1:**
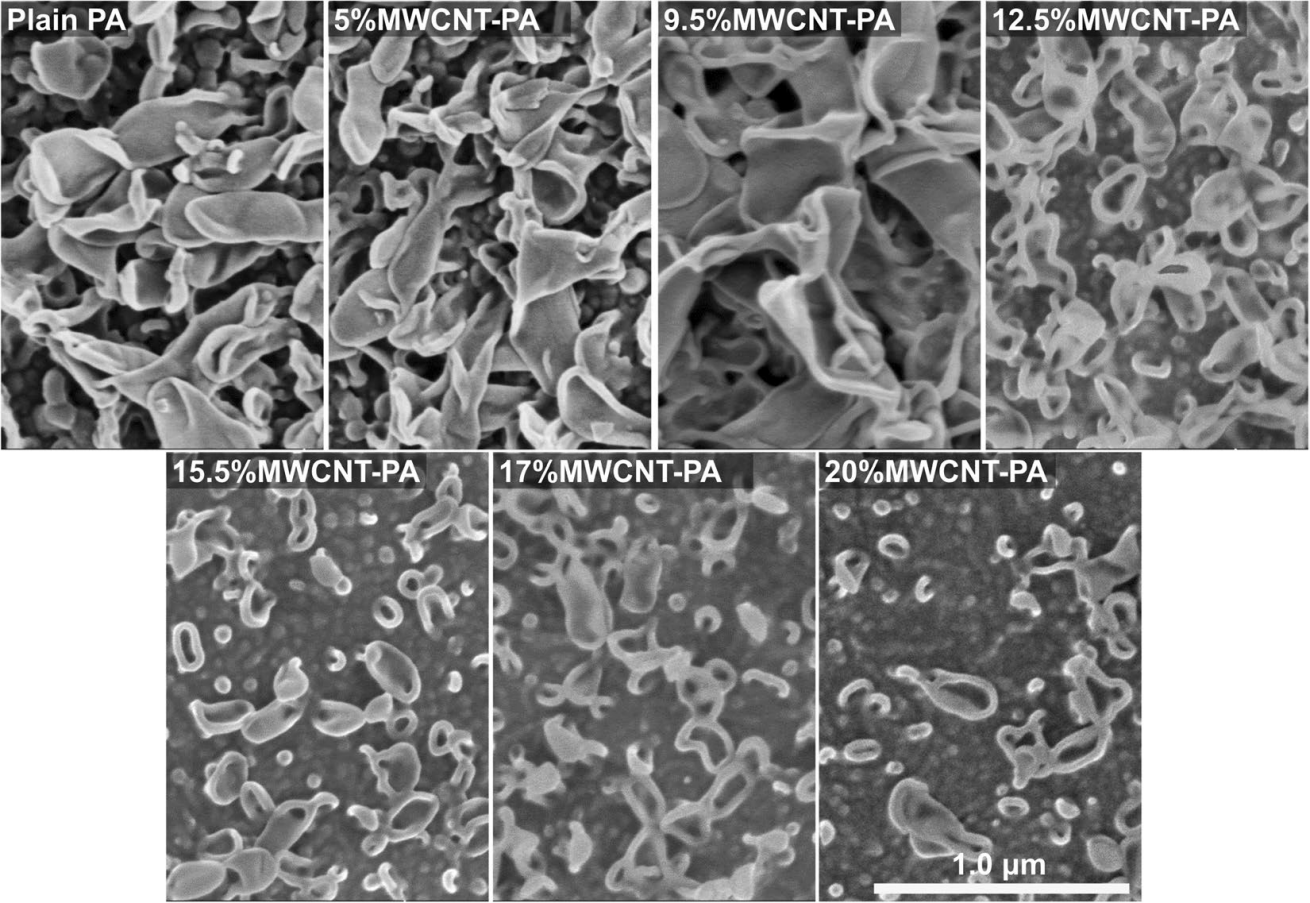
SEM images of MWCNT-PA nanocomposite membranes, for plain PA, and PA with 5, 9.5, 12.5, 15.5, 17 and 20 wt.% of MWCNT, where the typical lobe-like structures appear at the surface. Note the tendency towards a flatter membrane surface as the content of MWCNT increases. Scale bar corresponds to 1.0 µm for all the micrographs.

**Figure 2:**
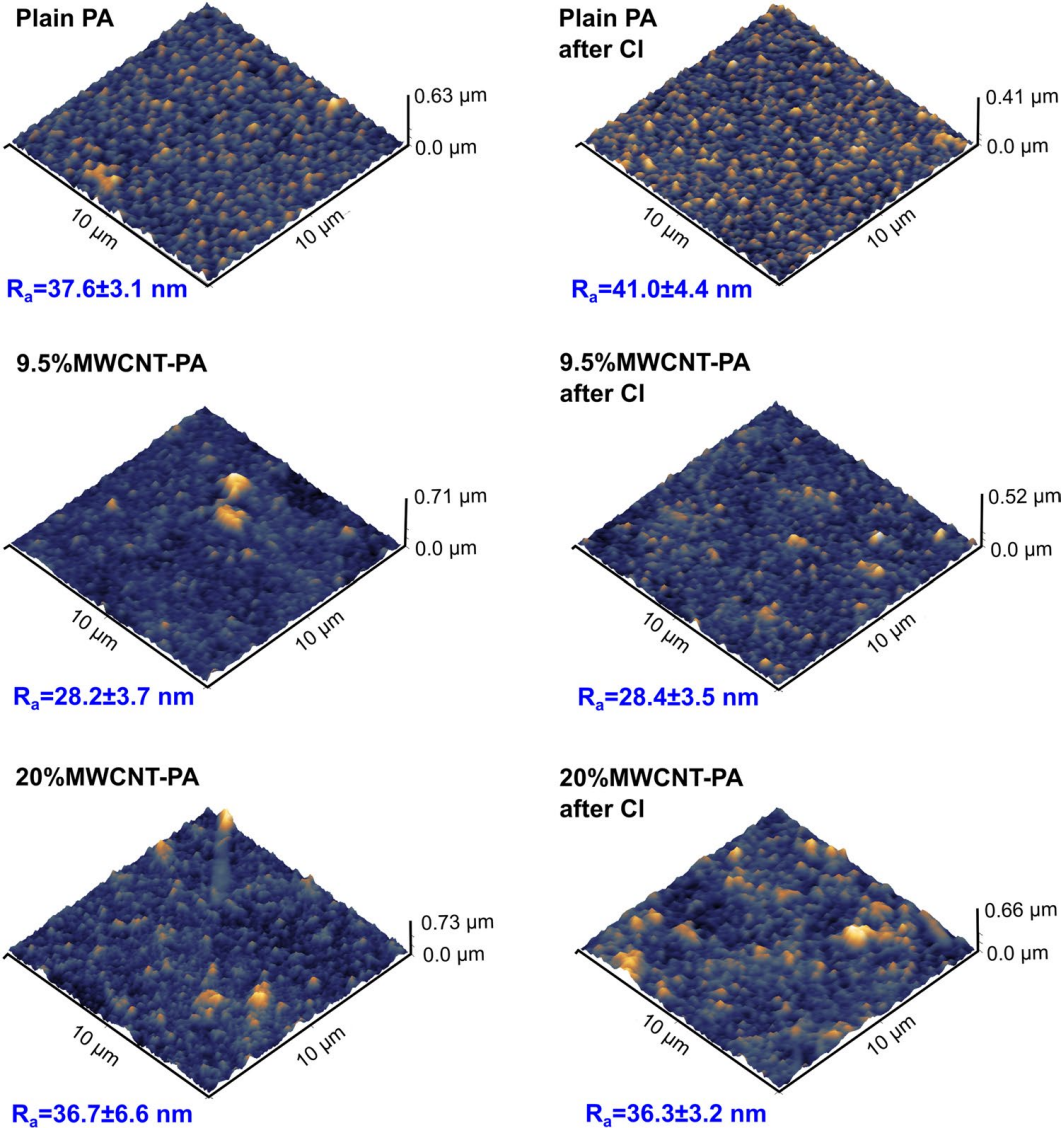
AFM images of MWCNT-PA nanocomposite membranes. The 3D views are shown for samples before (left) and after (right) chlorine exposure. From top to bottom the rows show samples of plain PA, 9.5 wt.% and 20 wt.% MWCNT-PA for analysis. The average roughness (*R*_*a*_) exhibits the different degradation behavior as a function of MWCNT content within the nanocomposite membranes.

The morphology of MWCNT-PA composite membranes was analyzed by SEM and AFM at different MWCNT content. Figure 1 shows SEM images of the membranes surface for samples prepared with different MWCNT loads. The MWCNT incorporation decreases the surface roughness of the active layer, resulting in a “flatter” surface. These morphological changes as a function of the carbon nanotube content have been addressed previously by other groups^3,17^, suggesting that the functional groups on the surface of nanotubes could interfere with the hydrogen bonding dynamics during the interfacial polymerization. In our case, the presence of surfactant functionalized nanotubes, that behave as a Pickering stabilizer, would also limit both diffusion and transport across the aqueous/organic solvent interface, by modifying the surface tension and interfacial polymerization process as it occurs with other organic solvents^18^. We used the average roughness (*R*_*a*_) calculated using the expression:

1 $${R}_{a}=\frac{1}{n}\sum _{i=1}^{n}|{y}_{i}|$$

where *y*_*i*_ is the height obtained from AFM measurements, (see Fig. 2), to assess the effects of chlorine exposure on the membrane surface. Overall, it can be observed that the *R*_*a*_ changes are almost negligible (variations smaller than 10 nm). However, it is necessary to stress that, from AFM characterization, it is not possible to observe a clear evidence of degradation induced by chlorine exposure, neither for the plain PA nor for the MWCNT-PA nanocomposite membranes, thus suggesting that the degradation processes occur at the nanostructure level. Figure S1 shows cross-sectional SEM images of membranes samples prepared with three different MWCNT concentrations: 5.0 wt.%, 15.5 wt.% and 20.0 wt%. The figure reports the average thickness from several measurements, demonstrating that the effect of MWCNT on the overall membrane thickness is negligible.

**Figure 3:**
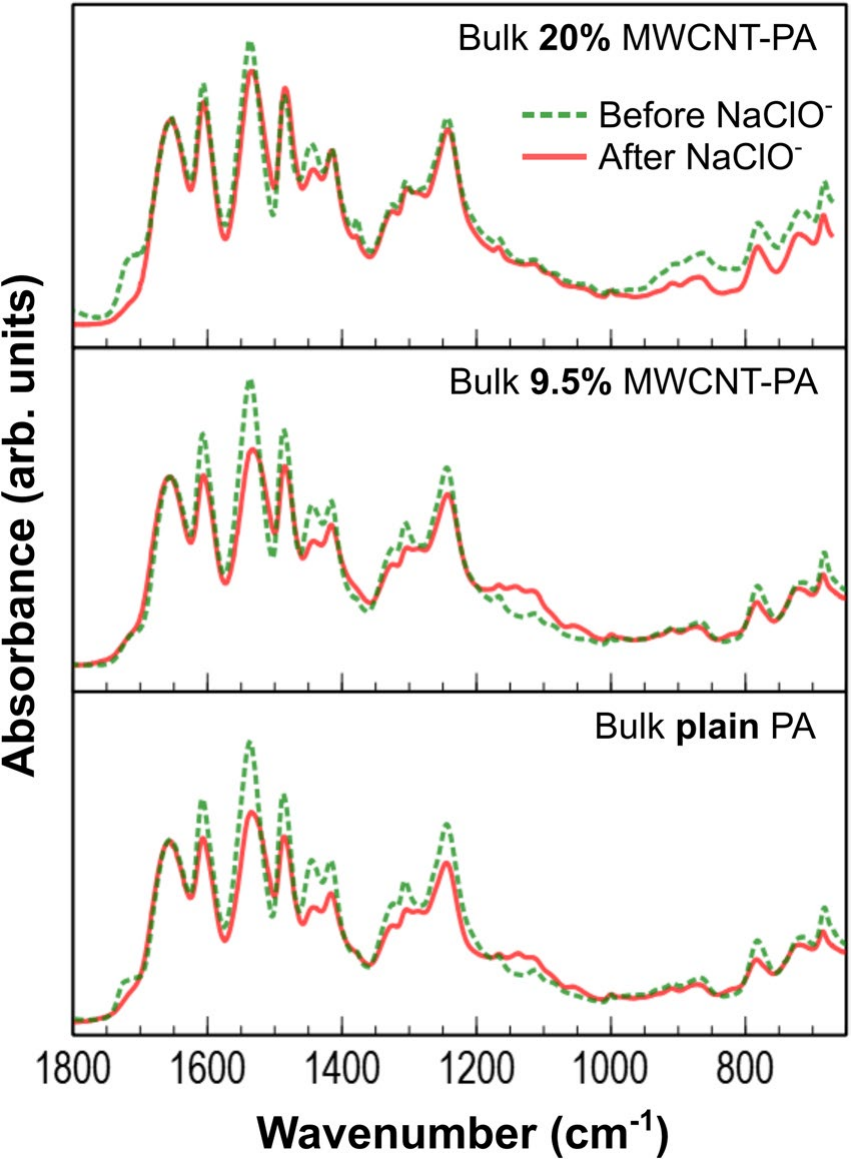
FTIR normalized spectra for bulk MWCNT-PA (plain PA, 9.5 wt.% and 20 wt.% of MWCNT) before (red solid lines) and after (green dashed lines) chlorine degradation tests. The spectra are superimposed to clearly identify chemical changes induced by ClO^−^ treatment.

The effects of chlorination of composite bulk samples were analyzed by FTIR (see Fig. 3), where normalized spectra are shown for plain PA, 9.5 wt.% and 20 wt.% MWCNT in PA. FTIR spectra indicated that ClO^−^ exposure promoted the N-chlorination of polyamide structure via electrophilic substitution, which has been previously suggested as the reversible first step of RO membrane degradation caused by chlorine species^7,8^. The plain PA and 9.5 wt.% MWCNT-PA spectra show the strongest decrease on both amide peaks intensity (aromatic around 1610 cm^−1^ and amide II band around 1540 cm^−1^). For the C-N stretching peaks (*ca*. 1240 cm^−1^ and 1300 cm^−1^) and the aromatic C=C stretching peak (*ca*. 1485 cm^−1^), the plain PA sample shows a slightly stronger variation than 9.5 wt.% nanocomposite. All the described changes in absorbance peaks suggest the cleavage of amide groups and chlorination of the PA rings. Also, the changes observed for the peak around 1720 cm^−1^ can be correlated with the quick chlorination of the polyamide ending groups, as reported previously^19^. The chlorination mechanism involves an Orton rearrangement followed by hydrolysis of the amide bond, as it has been discussed abundantly as a potential degradation mechanism of PA by chlorine^7,12^. In addition, the MWCNT-PA samples exhibit a more stable structure against chlorine degradation, and the peaks intensity scarcely changed when the MWCNT content was 20 wt.%. The FTIR spectra suggest that the degradation of MWCNT-PA is considerably lower than plain PA. Furthermore, FTIR analysis for MWCNT-PA membrane samples (see Figure S2a in Supplementary Information) did not show significant changes in terms of chlorine exposure, nevertheless, a useful feature was found: the intensity ratio of the aromatic C=C peaks (1485 cm^−1^/1505 cm^−1^) followed an empirical linear relationship which could be related to the MWCNT content on the composite membranes (see Figure S2b). These peaks are mainly produced by the PSU support layer, which is being detected by the IR radiation during measurements on the ultrathin MWCNT-PA nanocomposite layer. The fact that their ratio changes as a function of the nanotubes fraction, could be explained by a blocking effect of infrared radiation due to the presence of MWCNT within the membrane. The FTIR peak ratio of MWCNT content is useful for an empirical and practical assessment of the active layer composition in the range from 0 to 20 wt.% of MWCNT in the nanocomposite membranes.

**Figure 4:**
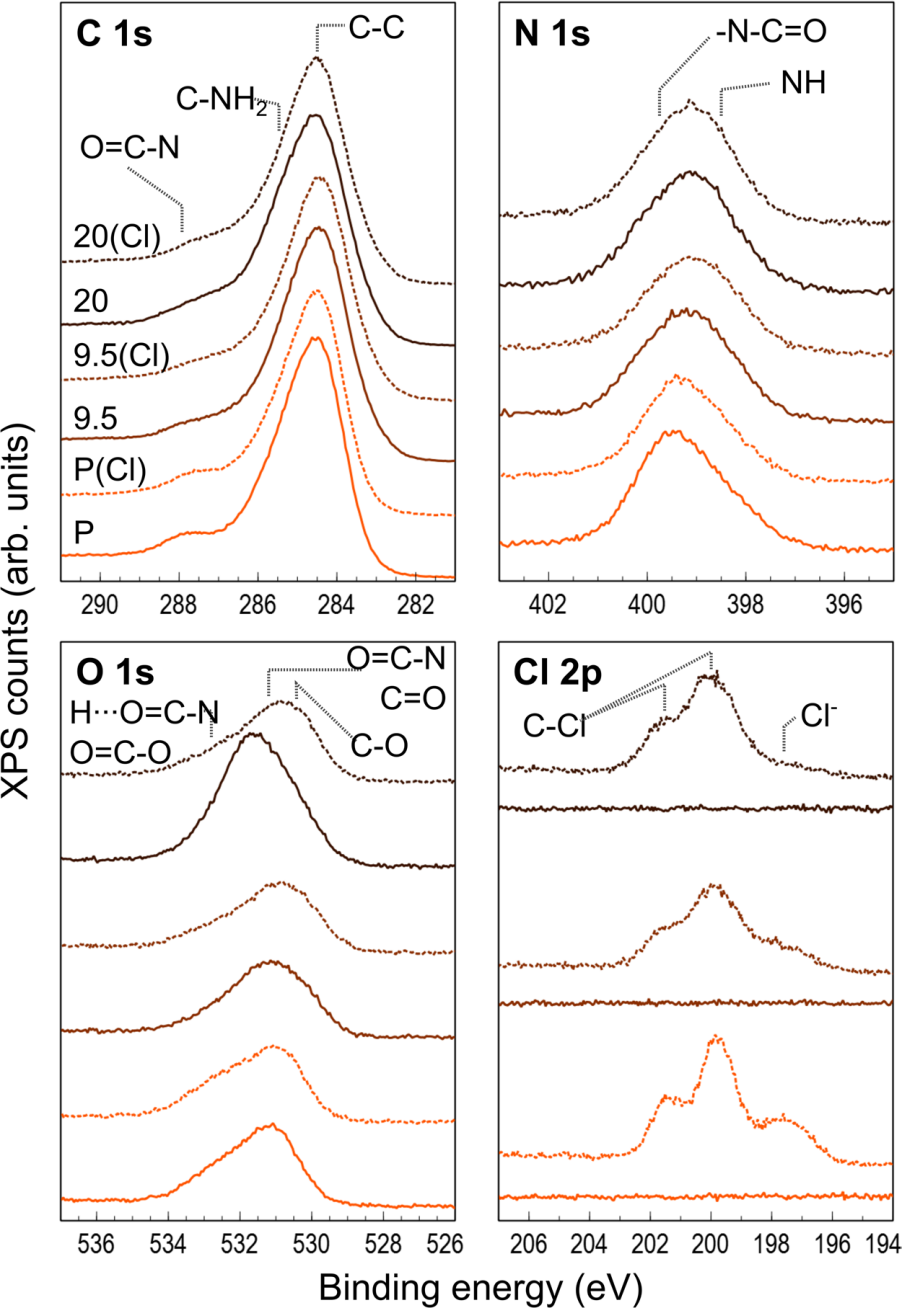
XPS core-level spectra of MWCNT-PA nanocomposite membranes. The spectra are shown for plain PA (P), 9.5 wt.% (9.5) and 20 wt.% (20) MWCNT-PA composite membranes, before (solid lines) and after ClO^−^ exposure (dashed lines). The scans correspond to C (1s), O (1s), N (1s) and Cl (2p) binding energies, with indications for key functional groups (mainly associated with PA and oxidation by chlorine species) binding energies.

XPS spectra of MWCNT-PA and plain PA membranes are shown in Fig. 4. Carbon, nitrogen, oxygen and chlorine were analyzed before and after chlorination, and the relative surface atomic composition was calculated and shown in Table 1. For chlorine quantification, only the covalent bond was considered, and the deconvoluted XPS peaks are shown in Figure S3. The elemental quantification of plain PA membrane samples matches the nitrogen and oxygen atomic content as reported elsewhere^8^, with values ranging between 9 and 12 at.% for nitrogen, and oxygen around 15 at.%. In addition, the XPS carbon scans reveal that the incorporation of MWCNT in fact reduces the quantity of C-O groups (see Fig. 4), which could arise from unreacted functional groups and would explain the increased stability of the PA matrix. Furthermore, for the MWCNT-PA composite membranes, a particular phenomenon was observed: whereas 9.5 wt.% MWCNT-PA membrane contains roughly the same proportion of nitrogen and oxygen (with a marginal but expected increase in carbon content from 72.5 at.% to 72.8 at.%), the 20 wt.% MWCNT-PA membrane shows a drastic increase in the oxygen content (21.6 at.%), specifically for the O=C-O groups observed in the O 1s signal. We attribute the higher oxygen content to the presence of residual anionic surfactant within the PA microstructure when high MWC NT loads are used, which would be completely removed after the membrane exposure to chlorine solution (note the change in O 1s XPS spectra for 20 wt.% MWCNT-PA before and after exposure to chlorine, Fig. 4). Moreover, the changes in the atomic proportions after exposure to chlorine reveals a clear protective effect of MWCNT within the composite: the Cl/N ratios for plain PA, 9.5 wt.% and 20 wt.% MWCNT-PA samples are 0.39, 0.32 and 0.33 respectively, thus demonstrating an effective content reduction of the amidic nitrogen chlorination.

**Table 1:** MWCNT-PA semiquantitative surface atomic composition from XPS quantification.

*Sample*	*C (at.%)*	*N (at.%)*	*O (at.%)*	*Cl (at.%)*
Plain PA	72.5 (70.0)	12.3 (10.4)	15.2 (15.4)	— (4.2)
9.5 wt.% MWCNT	72.8 (71.4)	12.3 (10.7)	14.9 (14.4)	— (3.5)
20 wt.% MWCNT	67.5 (68.3)	10.9 (11.7)	21.6 (16.0)	— (4.0)

The compositions are reported for membrane samples before and (after) ClO^−^ treatment.

Further analysis by positron annihilation lifetime spectroscopy (PALS) allowed a better description of the pore structural changes, produced by increasing the MWCNT content in the nanocomposite membranes. PALS provides information of pore size and associated free volume, by correlation with the positronium (electron-positron transitory bound state) lifetime. Ortho-positroniums (*o*-Ps), which are generated when electrons-positrons with parallel spin states combine, exhibit an intrinsic self-annihilation lifetime of 142 ns in vacuum, which can be shortened by their interaction with free electrons in pores within the structure they penetrate, usually polymeric thin films^20^. The pore size and pore quantity are related to the *o*-Ps lifetime (labeled τ_3_) by the Tao-Eldrup model, as described in the materials and methods section and elsewhere^21,22^. Figure S4 (in Supplementary Information) shows the measured positron lifetime for plain PA membranes, and for nanocomposite membranes with 9.5 wt.%, 15.5 wt.% and 20 wt.% load of MWCNT. Table 2 summarizes the deconvoluted τ_3_ (*o*-Ps) lifetime, its corresponding intensity (I_3_) and the calculated pore size (diameter in nm). It is noteworthy that, in terms of pore size, the PALS reveals negligible changes for an increasing amount of MWCNT within the nanocomposite, with variations in the range of 0.1 nm. Nevertheless, the intensity I_3_, which is correlated to the fraction of pores, decreases as the content of MWCNT increases up to 15.5 wt.%. The sample with 20 wt.% of MWCNT shows a marginal increase, which could be related to an overload of MWCNT within the PA matrix, thus affecting the fundamental structure of the PA chains. The anomalous result for the 20 wt.% sample can also be ascribed to a threshold value for MWCNT load at which the improvement effect on both salt rejection and water permeation stops (see below). Furthermore, the I_3_ and pore size of samples after chlorine exposure reveal an interesting phenomenon; the fraction of pores decreases for pure, 9.5 wt.% and 15.5 wt.% of MWCNT, whereas the pore size remains practically the same (largest increment equal to 0.1 nm). However, the intensity I_3_, which is correlated to pore fraction, decreases in agreement to previous reports describing the densification of the PA structure after chlorine exposure, followed by an increase in brittleness or compactability, depending on the original degree of PA aromaticity^23^. Again, the anomalous increase in I_3_ and pore size after chlorine exposure for the 20 wt.% sample suggests stronger degradation of the polymeric matrix produced by an excess of MWCNT.

**Table 2:** *o*-Ps lifetime (τ_3_), intensity and related pore size according with Tao-Eldrup model, for plain PA, and MWCNT-PA nanocomposite membrane samples.

Sample	τ_3_ (ns)		I_3_ (%)		Pore size (nm)	
	Before Cl	After Cl	Before Cl	After Cl	Before Cl	After Cl
Plain PA	1.885	2.043	11.0	7.4	0.5	0.6
9.5 wt.% MWCNT	1.922	2.036	10.5	5.1	0.6	0.6
15.5 wt.% MWCNT	1.958	2.024	4.8	3.1	0.6	0.6
20 wt.% MWCNT	2.025	2.310	6.0	13.1	0.6	0.7

The data was obtained from fitting the PALS spectrograms deconvolution (see Figure S4).

### Salt rejection and water flux evaluations

**Figure 5:**
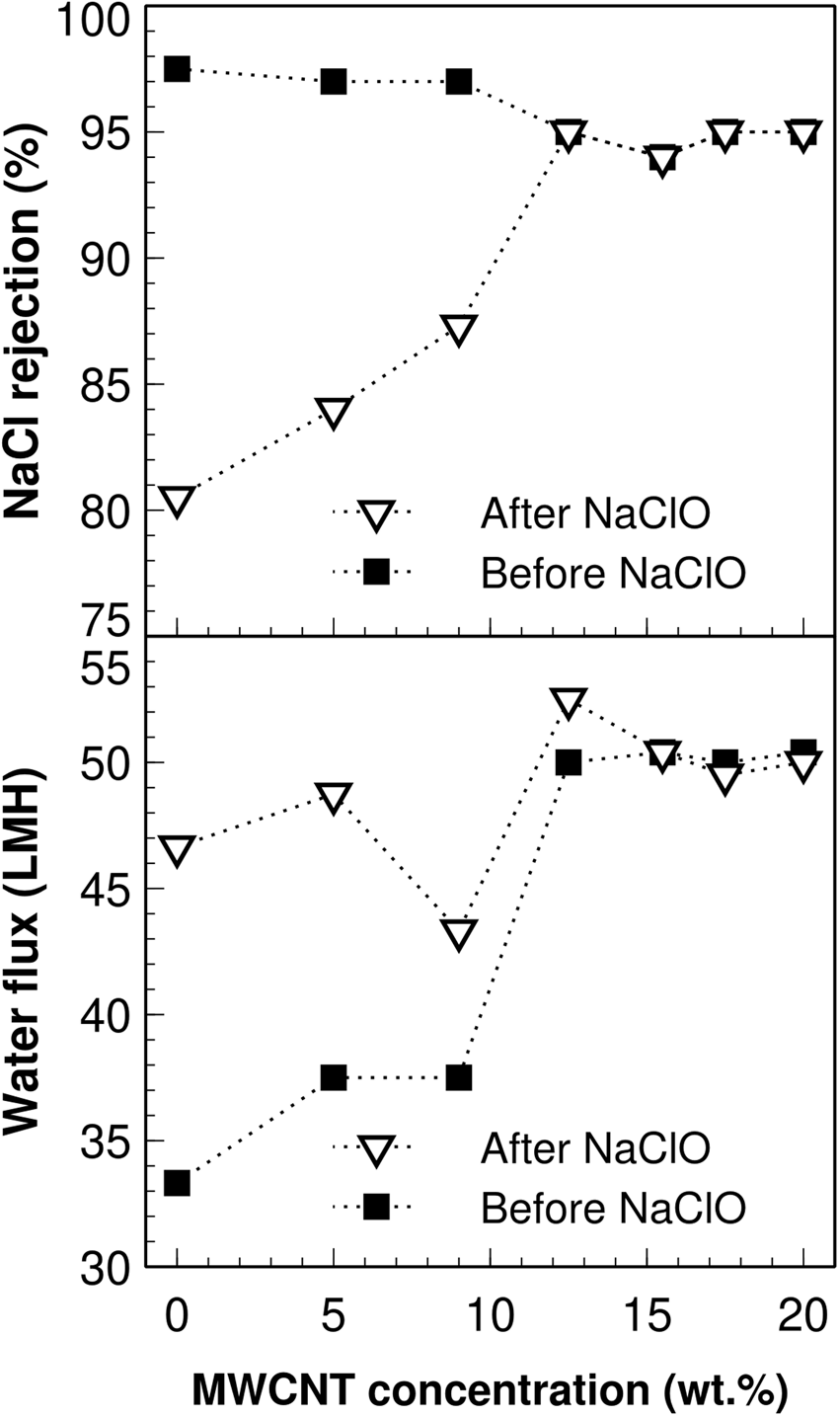
NaCl rejection and water flux performance for MWCNT-PA membranes. The performance was evaluated as a function of different MWCNT concentrations, before and after NaClO exposure (4800 ppm·h). Salt rejection and water flux changes after exposure to chlorine are drastically reduced with increasing MWCNT load. The membranes performance was evaluated in a cross-flow system operating at 5.0 MPa, with 3.5 wt.% NaCl solution.

The results for salt rejection and water flux before and after chlorine treatment as a function of MWCNT content are shown in Fig. 5. The chlorine resistance effect of MWCNT on the salt rejection and water flux is e vident for concentrations above 9.5 wt.% within the nanocomposites. Plain PA (0 wt.% of MWCNT) membranes perform better than membranes with high MWCNT content, nevertheless, after the chlorine treatment the salt rejection decreases drastically for this membrane, whereas the MWCNT-PA nanocomposite membranes retain their performance. Previous works^2,3^ have also reported the advantages of incorporating carbon nanotubes into PA matrices for RO membranes, highlighting the sustained salt rejection and water flux after chlorine treatments up to 3500 ppm·h (active chlorine capacity) for NaCl solutions of 0.2 wt.% during dynamic tests. However, the MWCNT-PA composite membranes analyzed here^14^ were exposed up to 4800 ppm·h of active chlorine, while using up to 3.5 wt.% NaCl solutions for separation tests, demonstrating an even higher chlorine resistance. Furthermore, Figure S5 in Supplementary Information shows additional long-term exposure tests to chlorine (up to 6000 ppm·h), that demonstrates the improved robustness of MWCNT-PA membranes against degradation. The performance improvement could be attributed to the relatively high content of MWCNT within the membranes, since most of the previous works utilize low loads of nanomaterials, typically less than 0.1 wt.%^2,3,24^.

The mechanistic nature of the observed chlorine resistance could be related to the previous studies reporting changes in the chemical activity (in some cases at electronic transfers level), experienced by different kind of molecules in proximity with *sp*^2^-hybridized carbon networks such as carbon nanotubes walls and graphene surfaces. For instance, it has been suggested^25^ that proteins such as glucose oxidase not only undergo structural rearrangements when adsorbed on to graphene surfaces, with an expected loss of activity, but also engage in electron transfers with the substrate. Moreover, the well-known phenomenon of graphene-based fluorescence-quenching is related to a charge transfer between aromatic molecules stacked by π-π interactions on top of *sp*^2^ hybridized carbon lattices, thus avoiding electronic excitations by providing stable electronic states^26^. Therefore, it could be hypothesized that the presence of MWCNT within the PA matrix has a stabilization effect expressed as reduced chemical activity for amide and other groups in the composite, thus making difficult their chlorination upon exposure to ClO^−^ ions. We further analyzed and explained the MWCNT-chlorine interaction by computational calculations, which will be addressed below.

It is interesting to note the threshold value of the MWCNT fraction embedded within the composite membrane at which the performance of the membrane is effectively improved (in this case starts when having 12.5 wt.% of nanotubes). Specifically, the water flux before chlorine treatment was significantly increased, going from 33.5 to around 50 L m^−1^ h^−1^ (LMH), with a negligible change in the salt rejection. The experimental increment of water flux contrasts with our previous theoretical results obtained by molecular dynamics stating that the actual water diffusion path is increased marginally for an increasing amount of SWCNT load in PA matrixes, thus decreasing the water flux^15^. A similar effect has been reported experimentally for membranes of carbon nanotubes^3^ and graphene oxide^24^, where higher water flux is obtained at higher carbon nanomaterial loads (from *ca*. 15 to 27 LMH using MWCNT, and from 34 to 62 LMH using GO, respectively). The high-permeation could be explained by a percolation-hopping model for water as explained elsewhere^27^, which proposes the creation of hydrophilic pockets within the locally densified PA structure due to the presence of MWCNT, facilitating new low-energy paths for water molecules in the proximities of MWCNT. This new model complements others that have been proposed as a mechanism for improved water permeation through graphene-based membranes^28^.

### Molecular simulations

**Figure 6:**
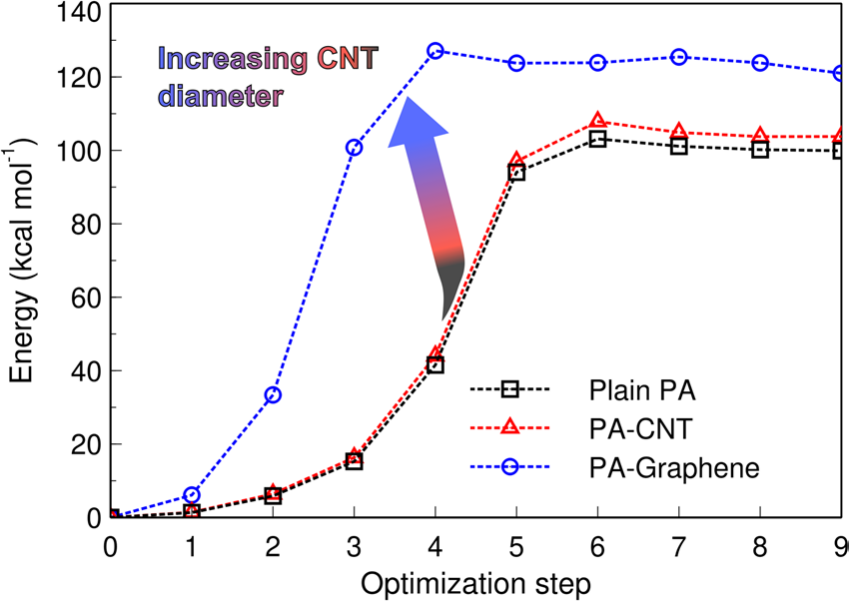
Energy plot for the different optimization steps along the NEB routine. The activation energy for each case is plotted; plain PA (black squared marks), PA in vicinity of a CNT (red triangle marks) and PA in vicinity of a graphene section (blue circle marks). The PA-Graphene system resulted in a higher activation energy for chlorination of PA moieties, which relates to the higher resistance of nanocomposite MWCNT-PA membranes to degradation by chlorine exposure.

We used quantum/first principles calculations to assess the effects of MWCNT addition to the PA membrane. The NEB method was used to calculate the activation energy of the amide chlorination step, in presence or absence of a vicinal *sp*^2^-hybridized carbon network. Figure 6 shows the activation energy from NEB calculations, as a function of time in terms of optimization steps. The chlorination energy barrier profiles for plain PA and for PA in proximity of a (5,5) CNT are similar for the NEB calculation, being both lower in energy (about 20 kcal mol^−1^) than the system with PA in proximity of a graphene fragment. The higher activation energy indicates that graphene provides a more effective barrier against chlorination for PA moieties, within the particular model we studied. A possible explanation could be related to effects exerted by carbon nanomaterials curvature on surrounding molecules, which already has been addressed theoretically and experimentally as a key parameter for carbon nanomaterials reactivity^29,30^. In this case, graphene can also be regarded as a model for a large diameter CNT surface, which is the case for the MWCNT added to the RO composite membranes (average diameter *ca*. 10 nm). The interaction of graphene with adsorbed moieties can exhibit a wide variety of behaviors, as has been reported in the literature^31,32^. Given the current understanding of adsorption phenomena on graphene, we believe that π-π stacking dominates the interaction between *sp*^2^-hybridized lattices and PA aromatic molecules^15^, and it affects the chemical activity of PA in the proximity of MWCNT.

**Figure 7:**
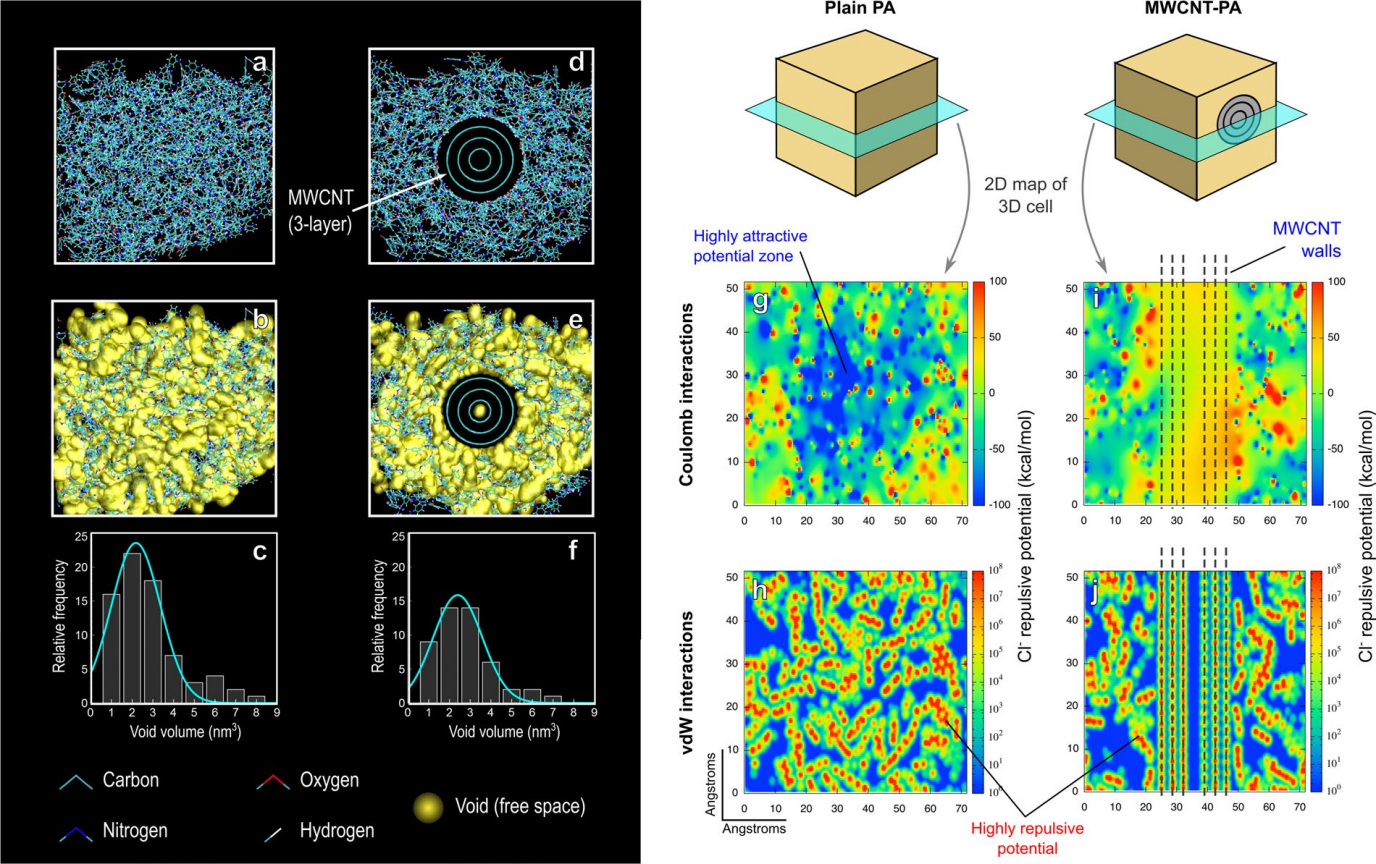
Simulation results for PA and MWCNT-PA molecular models. Figures on the left-side show analysis for free space changes: (**a**,**b**) show plain PA models, and (**d**,**e**) MWCNT-PA models with/without the spatial representation of voids. (**c**,**f**) show the voids (free space pockets) volume distribution, as found by the parameters used. The voids volume distribution reveals an expected decrease, given the high density of PA matrix surrounding the MWCNT. On the righthand side, the figures show repulsive potential maps for Cl^−^ ion, for (**g**,**h**) plain PA and (**i**,**j**) MWCNT-PA cells used for molecular dynamics simulations (**a**,**d**). The potential maps are shown for long-range Coulomb interactions (top maps), and short-range vdW interactions (bottom maps), where positive (red) values represent repulsive potential, whereas negative (blue) values represent attractive potential. The potential maps reveal the “closing” effect produced by MWCNT in the PA matrix, by increasing the regions with repulsive potential for Cl^−^ ions.

The presence of small pores and channels (0.4 to 1 nm) is well-known to affect water and ion diffusion within the PA membrane^33^, so we quantified these voids as reported before for carbon based membranes^34^, and the results are shown in Fig. 7a–f. The void-size distribution indicates that the free-volume fraction in the presence/absence of a MWCNT results in a *ca*. 9% higher free-volume for plain PA when compared with MWCNT-PA composite structure. This result is similar to the PALS measurements on MWCNT-PA nanocomposite membranes, and would be expected because any nanostructure like MWCNT embedded in the polymer matrix would produce high density regions around it due to strong vdW forces. Indeed, the higher density matrix of polyamide in our previous study was partially responsible of a slight decrease in the water diffusion^15^. As mentioned above, the experimentally higher permeation can be explained by an improved water percolation path, produced by local changes in density due to the presence of MWCNT.

Finally, Fig. 7g–j also shows the calculated repulsive potential for Cl^−^ ions, against the MWCNT-PA and the plain PA cases. The potentials are depicted as 2D maps, taken from the middle section of each molecular model (Fig. 7a,d). The repulsion potential maps reveal an effect related to an increased density of the polymeric matrix around the nanostructures, which can result from a reduction of non-repulsive Coulomb interaction regions (see blue regions on Fig. 7g,i). This densification of the matrix decreases the penetration of solvated ions and other species into the PA structure, thus improving the structure stability and rejection performance.

## Conclusions

Nanocomposite MWCNT-PA membranes exhibit high chlorine resistance when immersing them in aqueous solutions of NaClO. The mechanism was studied by structural characterization of the membranes exposed to relatively high concentrations of chlorine solutions. The experimental results indicate that the presence of MWCNT within the PA matrix in composite membranes alter significantly both the surface morphology (i.e., surface shape and roughness), and the molecular topology. Spectroscopic analyses reveal lower degradation of the PA matrix, which, was also supported by molecular simulations. These results are attributed to a protective effect of MWCNT that act as PA anti-chlorination stabilizers. The higher chemical stability of the nanocomposite arises from an increased activation energy for chlorination. This stability is induced by interactions between PA and graphene-like *sp*^2^-hybridized carbon networks. In addition, the changes in density of the PA matrix produced by the incorporation of MWCNT seems to reduce the chlorine uptake, which would contribute to the reduced degradation by chlorine exposure.

Regarding water desalination tests, when increasing the MWCNT content of the nanocomposite membranes, an increased water flux and a slight decrease in salt rejection were observed. Moreover, their performance after static treatments with chlorine aqueous solutions demonstrated enhanced resistance against degradation when compared to plain PA RO membranes. MWCNT-PA composite membranes are therefore a feasible and competitive alternative to improve PA membrane performance for their use in RO water desalination systems with an expected longer lifetime when compared to plain PA membranes.

## Materials and Methods

### Membrane preparation

1,3-diaminobenzene (>98%) (MPD) and 1,3,5-benzene tricarbonyl trichloride (>98%) (TMC) were acquired from Tokyo Chemical Industry Co. Inc. Hexane (>96%), sodium chloride (>99.5%) (NaCl), and sodium hypochlorite solution (5.0 wt.%) (NaClO) were purchased from Kanto Chemical Co., Inc. (Purity > 96%). All other reagents were of analytical grade or better. An aqueous anionic dispersion of MWCNT (Nanocyl NC-7000, Japan) was prepared as reported elsewhere^35^. Polypropylene nonwoven supported porous polysulfone (PSU) membranes (cutoff Mw = 100 000) were acquired from *Alfa Laval DSS* (Cat. No. GR40PP). In a typical membrane synthesis, PSU support membranes were soaked in 2.0 wt.% MPD aqueous solution for 3 h and subsequently soaked in TMC/hexane solution (0.1 wt.%) for 2 min. The membranes were subsequently dried at room temperature for several hours. For the MWCNT containing PA membranes, MPD aqueous solutions containing a variable amount of dispersed MWCNT stabilized by an anionic surfactant were used (see Figure S6 for a scheme of the membrane preparation). Table S7 relates the final wt.% fraction of MWCNT with the MWCNT:H_2_O ratio of dispersions used for the membrane preparation. The final MWCNT fraction was determined by TGA of membrane samples (see Figure S8).

### Membrane characterization

The micro-structure of MWCNT-PA composite membranes was analyzed by scanning electron microscopy (SEM, Hitachi SU8000 FE-SEM, Tokyo, Japan) and atomic force microscopy (Agilent Technologies AFM 5500, Santa Clara, CA, USA). Topography images were acquired and analyzed using the Gwyddion^36^ software. X-ray photoelectron spectroscopy analysis was performed using the Al-Kα line in an XPS system (PHI Quantera II XPS Microprobe, Physical Electronics, Chanhassen MN, USA), on *in-situ* etched samples (2 nm depth, Ar ion gun). Semi-quantitative chemical analysis was performed by integrating the C 1s, O 1s, N 1s and Cl 2p peaks from XPS survey scans, and the samples were referenced to the C 1s *sp*^2^ peak at 284.5 eV. Deconvolution of chlorine signal was carried out to resolve the covalent bond from the adsorbed chloride. The Cl 2p signal was deconvolved into doublet peaks due to its 2p1/2 and 2p3/2 spin-orbit splitting. The area ratio for the two spin-orbit peaks (2p1/2:2p3/2) was kept constant at 1:2 ratio with a peak separation of 1.6 eV. All FWHM within the same spectrum were kept constant. The signal with higher binding energy correspond to the covalently bonded chlorine while that of lower binding energy to the adsorbed chloride anion. Fourier-transform infrared spectroscopic studies (Thermo Scientific Nicolet 6700 FT-IR, Waltham, MA, USA) were performed in attenuated total reflectance (ATR) mode on bulk and in membrane samples. For the bulk samples spectra, the baseline was removed automatically using the on-system tool for baseline subtraction, and the signal intensities were normalized using the peak located at *ca*. 1658 cm^−1^. Thermogravimetric analysis was conducted on a TGA 8120 apparatus under flowing humidity-controlled filtered air (300 mL min^−1^) and at a heating rate of 10 °C min^−1^. For PALS measurements, a depth-selective positron nano-porosimetry system (PALS 200A, Fuji Imvac Inc, Yokohama, Japan) was used, with a Na-22 as a positron source and 1 keV beam energy. The membrane samples (PA or MWCNT-PA active layer on PSU porous substrate) were cut into 15 mm × 15 mm approximately, and vacuumed at values lower than 10^−6^ Pa during measurements. The free volume radius was calculated using the Tao-Eldrup model^21,22^, expressed as:

2 $${\tau }_{3}=0.5{[1-\frac{R}{R+0.1656}+\frac{1}{2\pi }sin(\frac{2\pi R}{R+0.1656})]}^{-1}$$

with *τ*_3_ (ns) fitted from the acquired PALS spectra as the *o*-Ps pick-off annihilation lifetime, and *R* being the radius of positron trapping site, equivalent to free volume.

### Chlorine resistance tests

Composite membrane chlorine degradation was evaluated by static exposure tests described as follows: first, membrane samples were immersed in 200 ppm NaClO solution in distilled water with pH *ca*. 9.0 at room temperature during 24 h. The chlorine concentration was constantly monitored (using an Ultra High Range Chlorine checker, HANNA Instruments, RI, USA), and kept at 200 ppm during the experiments. After the predetermined immersion time, the membranes were washed in distilled water and 1000 ppm sodium hydrogen sulfite aqueous solution was used to remove any remaining chlorine. Subsequently, water permeation and salt rejection performances were evaluated for treated membranes using a laboratory scale cross-flow system like the one previously reported^34^, in which circular membranes with effective area of 2.27 cm^2^ were placed in a stainless-steel unit cell. The cross-flow tests were carried out using 5.0 MPa for transmembrane pressure, while maintaining a feed water flux *ca*. 300 mL min^−1^, which corresponds to a water velocity at the membrane surface of 0.27 m sec^−1^. The tested saline water concentration was 35000 ppm of NaCl (corresponding to 3.5 wt.%). For permeated water, flux and salt rejection measurements were carried out by measuring permeate volume per time unit, and by measuring permeate conductivity (Horiba Scientific LAQUAact ES-71 conductivity meter, Kyoto, Japan) respectively. Additional long-term tests for chlorine resistance were carried out for membranes with 15.5 wt.% MWCNT and plain PA, exposing them to 20 ppm NaClO solutions for up to 300 hrs., under dynamic test, which consists in cross-flow operation using 0.2 wt.% NaCl solution plus the NaClO.

### Molecular simulations

The activation energy barrier for chlorination of PA was studied by Nudged Elastic Band (NEB) calculations using three structural models: 1) plain PA, 2) SWCNT (5,5) + PA, and 3) graphene + PA, in order to evaluate the effect of *sp*^2^ hybridized carbon networks on the PA stability. The PA structure was built by one TMC and two MPD molecules, 44 atoms in total. The SWCNT (5,5) and graphene were both composed of 100 atoms. We performed structural relaxation for the three cases followed by the NEB20 simulations, which were performed by the *Quantum Espresso* package, considering van der Waals (vdW) interactions^37^. During simulations, a plane wave basis set with 30 Ry cutoff energy and ultra-soft potential within PBE^38^ as exchange correlation interaction functional was considered, and the reaction path was set to ten images. For NEB simulation, we set the amide bond of PA + free chlorine in the initial state and the amide bond attached chlorine + free hydrogen atoms in final state. During NEB calculations, the initial and final states were also optimized by performing structural relaxation.

Molecular dynamics on model PA and PA-CNT membranes were carried out as reported in the literature^15^. Briefly, a PA matrix was simulated as interacting monomers (1,3-diaminobenzene or MPD, and 1,3,5-benzenetricarbonyl trichloride or TMC), using Lennard-Jones (LJ) and Coulomb type potentials for energy evaluations. Parametrized atom charges were previously determined with *ANTECHAMBER* 1.27 and AM1-BCC partial charges^39,40^. Interactions between carbon atoms from CNT and PA chains are determined by LJ only, as parametrized in the GAFF (force field). CNT interactions are described by the Tersoff potential^41^ and LJ potential among layers with cutoff radii set to 10.0 Å. For plain PA, a cell of 71.8 × 51.4 × 80.1 Å was filled with a PA molecule made with 360 and 282 MPD and TMC units respectively; isothermal-isobaric (NPT) ensemble relaxed for 2 ns, with a time step of 0.5 fs. For CNT-PA, the cell size was preserved, while reducing the number of PA molecules made with 291 and 228 MPD and TMC units, in order to maintain a constant density. The CNT was triple-walled [(15,15) > (10,10) > (5,5)] with a total of 2520 carbon atoms. Additionally, free volume (voids) determinations within the relaxed PA and CNT-PA cells were carried out with the *Fpocket* code^42^, using probe spheres between 3.0 and 6.0 Å, 50 spheres as minimum clustering, and 4 neighboring spheres as interclustering criteria for free volume detection. Finally, 2D maps of potential for Cl^−^ ions were calculated using:

3 $${V}_{i}=\sum _{j}4{\varepsilon }_{ij}[{(\frac{{\sigma }_{ij}}{{r}_{ij}})}^{12}-{(\frac{{\sigma }_{ij}}{{r}_{ij}})}^{6}]+\sum _{j}\frac{{q}_{i}{q}_{j}}{4\pi {\varepsilon }_{0}{r}_{ij}}$$

which defines the interaction potential *V*_*i*_ as a sum of vdW and Coulomb potentials. The first term is vdW interaction evaluated by LJ potential, and the second term is the Coulombic interaction. LJ parameter *ε*_*i*j_ was 0.10 kcal mol^−1^ and *σ*_*i*j_ was set to 4.40 Å for Cl. Charges of Cl, *q*_*i*_, are −1.0^43^. The LJ interaction potentials for other atoms combinations were determined by Lorentz-Berthelot combination rules. The potential mapping surface for Na and Cl was calculated in the XY plane centered along the Z direction.

## Electronic supplementary material

Supplementary Information
