# Supplementary material for: Robust water desalination membranes against degradation using high loads of carbon nanotubes
Source: Sci Rep. 2018 Feb 9;8:2748. doi: 10.1038/s41598-018-21192-5 (PMC5807517; doi:10.1038/s41598-018-21192-5)
Supplement: Supplementary file 1 — Supplementary Information [file 41598_2018_21192_MOESM1_ESM.pdf]

Robust water desalination membranes against degradation using high loads of carbon nanotubes

SUPPLEMENTARY INFORMATION

J. Ortiz-Medina[1*], S. Inukai[1], T. Araki[1,2], A. Morelos-Gomez[1], R. Cruz-Silva[1,3], K. Takeuchi[1,3], T. Noguchi[3], T. Kawaguchi[3], M. Terrones[3,4] and M. Endo[1,3*]

[1] *Global Aqua Innovation Center, Shinshu University, Nagano 380-8553, Japan.*

[2] *Division of Computational Science and Technology, Research Organization for Information Science and Technology, Tokyo 140-0001, Japan.*

[3] *Institute of Carbon Science and Technology, Faculty of Engineering, Shinshu University, Nagano 380-8553, Japan.*

[4] *Department of Physics, Department of Chemistry, Department of Materials Science and Engineering & Center for 2-Dimensional and Layered Materials, The Pennsylvania State University, PA 16802, USA.*

*Email: ortizmed@shinshu-u.ac.jp; endo@endomoribu.shinshu-u.ac.jp

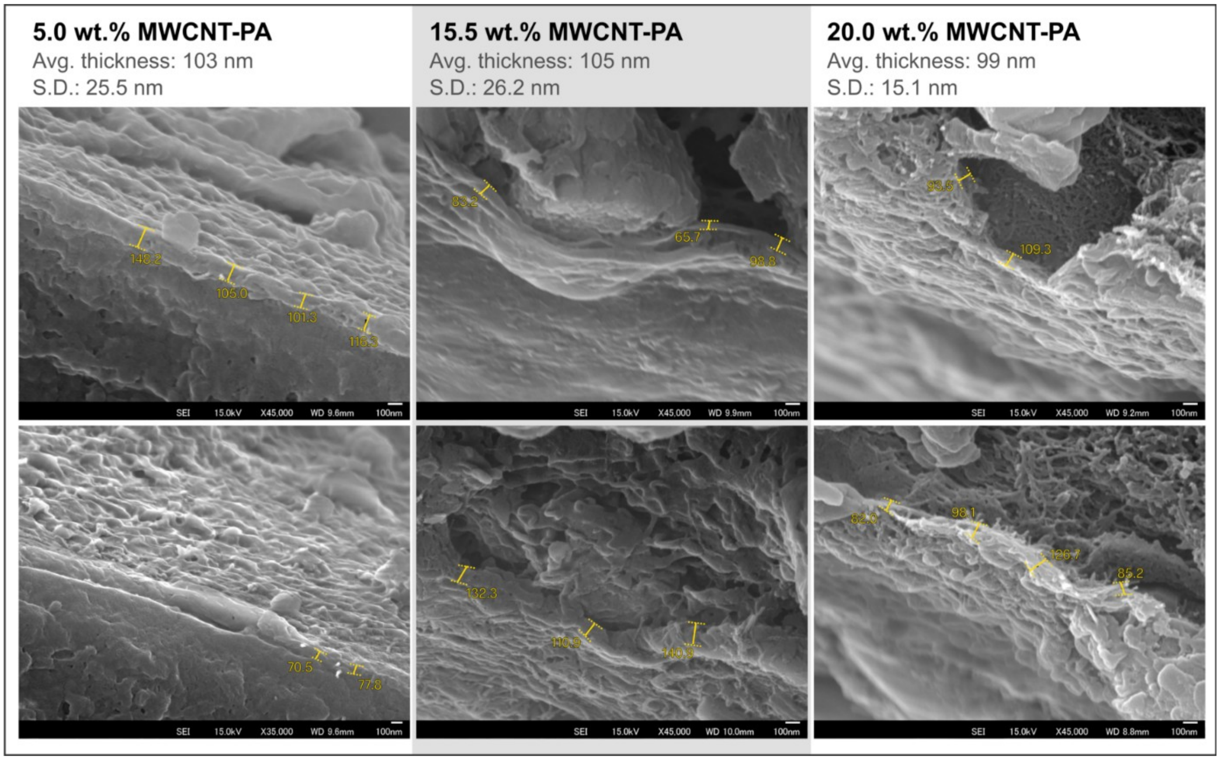

Figure S1. Cross-section SEM images of MWCNT-PA nanocomposite membranes. Two images are shown for nanocomposite membranes made using 5.0, 15.5 and 20.0 wt.% of MWCNT. Thickness measurements indicate a negligible effect of MWCNT concentration in the average thickness of the membranes.

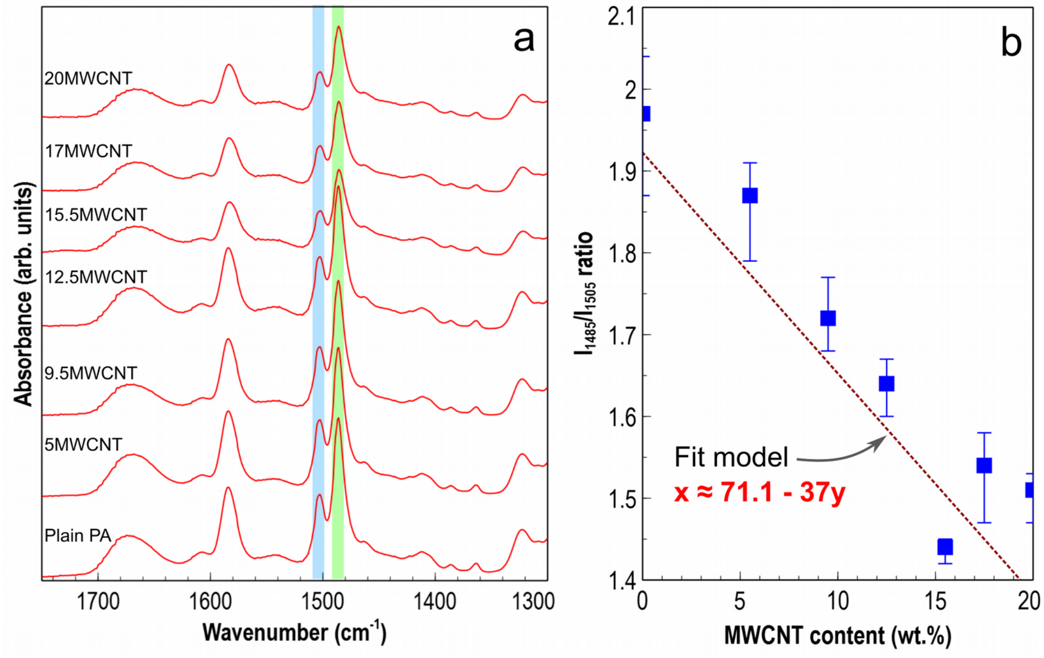

Figure S2. MWCNT concentration by FTIR. a) FTIR spectra for membrane samples (no chlorine exposure), where all MWCNT concentrations were analyzed. The peaks from aromatic carbons (*ca*. 1485 and 1505 cm^{-1}) are highlighted, and their ratios are plotted in b), where an empirical model has been found in function of MWCNT content.

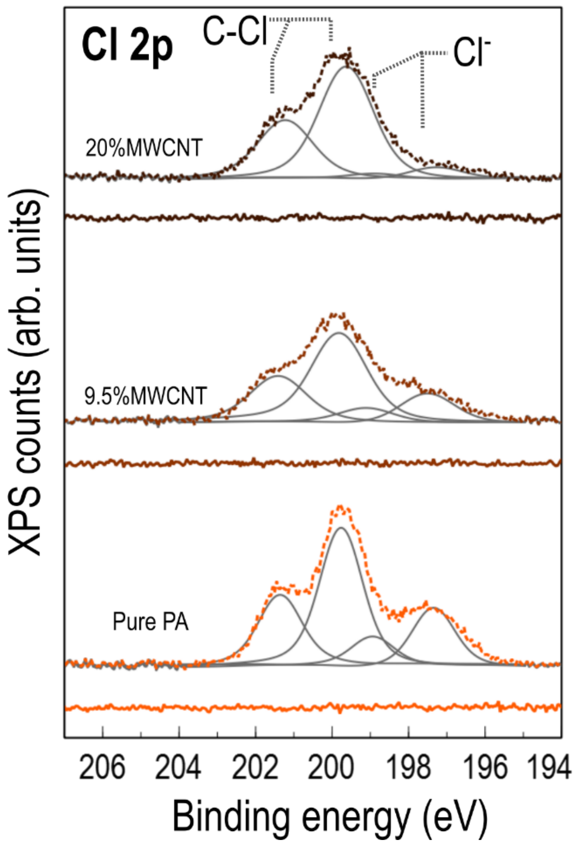

Figure S3. Deconvoluted chlorine XPS core-level spectra. The plot depicts spectra for plain PA, 9.5 wt.% and 20 wt.% of MWCNT in nanocomposite membranes. The spectra have been deconvoluted for discerning of covalently bound and adsorbed chlorine species. Only covalently bound Cl has been considered for quantification (chlorination) within MWCNT-PA nanocomposites.

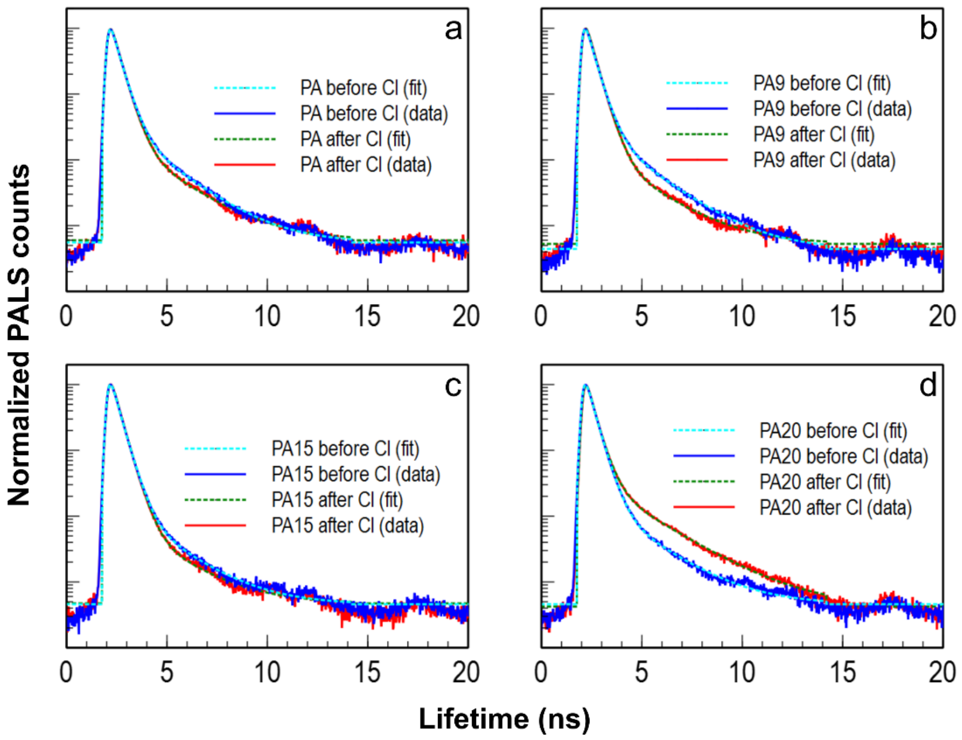

Figure S4. Positron lifetime spectra. a) plain PA, b) 9.5 wt.% MWCNT-PA, c) 15.5 wt.% MWCNT-PA and d) 20 wt.% MWCNT-PA nanocomposite membranes. Each plot shows the measured data and its corresponding fit (up to τ3) for membrane samples before/after exposure to chlorine. The deconvoluted τ3 lifetime, intensity and associated pore size were calculated and reported on table 2 (main manuscript).

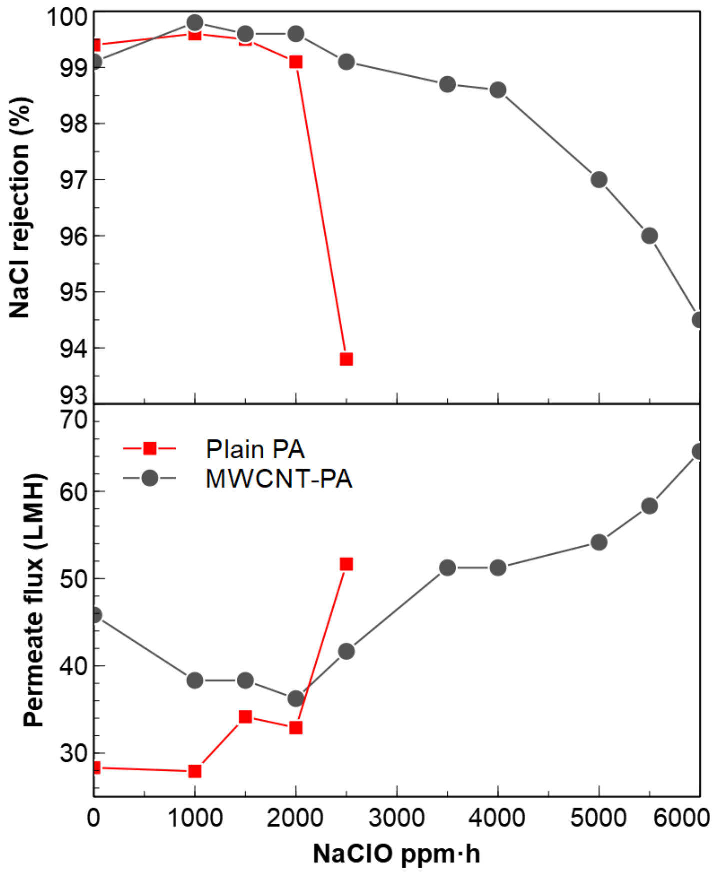

Figure S5. Long chlorine exposure test. Chlorine resistance tests up to 6000 ppm·h were carried out for plain PA membrane (Plain PA) and 15.5 wt.% MWCNT-PA membrane (MWCNT-PA), for comparing their performance after long term exposure. The plot shows the evolution of salt rejection and permeability, during a cross flow test operating with 0.2 wt.% NaCl saline solution, 20 ppm NaClO and under 0.75 MPa as operating pressure.

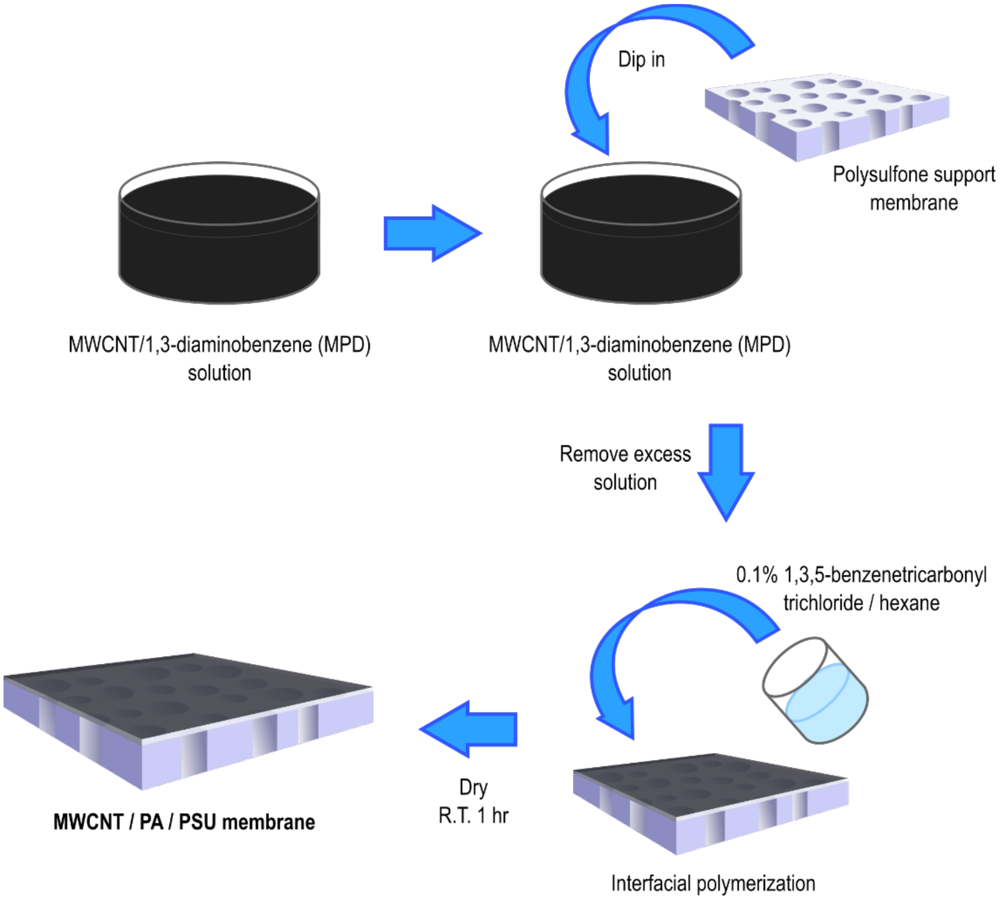

Figure S6. Scheme of MWCNT-PA membrane synthesis. The fabrication of MWCNT-PA nanocomposite membranes consists in soaking of PSU support membrane in a MWCNT-MPD solution, followed by interfacial polymerization with TMC and drying at room temperature.

Table S7. MWCNTs dispersions vs final MWCNT fraction in membranes. MWCNT content in the aqueous solutions used for MWCNT-PA composite membranes fabrication (left column) related to the final MWCNT content within nanocomposite membranes (right column). All samples were produced using a constant 2.0 wt.% of MPD in solution.

**MWCNT:H_2O ratio (wt.%)**	**Final MWCNT content (wt.%)**
0.00 : 98.00	0 (plain PA)
0.10 : 97.90	5.0
0.20 : 97.80	9.5
0.30 : 97.70	12.5
0.40 : 97.60	15.5
0.45 : 97.55	17.0
0.50 : 97.50	20.0

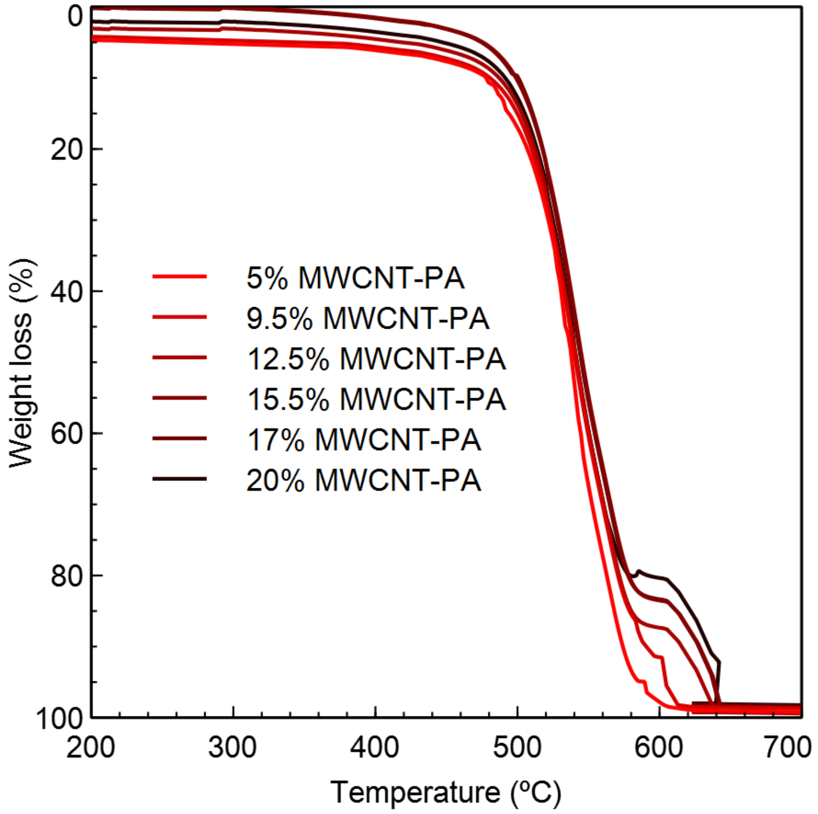

Figure S8. TGA of MWCNT-PA membranes. The analysis was done for final MWCNT fraction determination, given the parameters for membrane synthesis shown in table S4. The final MWCNT fraction was derived from the point where the abrupt change in thermal degradation profile occurred for each sample (in between 580 and 640 °C).
